# The relevance of timing of illness and death events in the household life cycle for coping outcomes in rural Uganda in the era of HIV

**DOI:** 10.1186/s12939-015-0253-0

**Published:** 2015-10-27

**Authors:** Jovita Amurwon, Flora Hajdu, Janet Seeley

**Affiliations:** Department of Urban and Rural Developmen, Swedish University of Agricultural Sciences, Box 7012, SE-750 07 Uppsala, Sweden; Centre for International Health, University of Bergen, Box 7804, N-5020 Bergen, Norway; Medical Research Council/Uganda Virus Research Institute, Research Unit on AIDS, Box 49, Entebbe, Uganda; London School of Hygiene and Tropical Medicine, London, UK

**Keywords:** Age, Capability, Dependency-ratio, Diversification, Livelihood, Longitudinal studies, Shock, Struggling, Coping

## Abstract

**Introduction:**

Predicting the household’s ability to cope with adult illness and death can be complicated in low-income countries with high HIV prevalence and multiple other stressors and shocks. This study explored the link between stage of the household in the life cycle and the household’s capacity to cope with illness and death of adults in rural Uganda.

**Methods:**

Interviews focusing on life histories were combined with observations during monthly visits to 22 households throughout 2009, and recorded livelihood activities and responses to illness and death events. For the analysis, households were categorised into three life cycle stages (‘Young’, ‘Middle-aged’ and ‘Old’) and the ability to cope and adapt to recorded events of prolonged illness or death was assessed.

**Results:**

In 16 of the 26 recorded events, a coping or struggling outcome was found to be related to household life cycle stage. ‘Young’ households usually had many dependants too young to contribute significantly to livelihoods, so were vulnerable to illness or death of the household head specifically. ‘Middle-aged’ households had adult children who participated in activities that contributed to livelihoods at home or sent remittances. More household members meant livelihood diversification, so these households usually coped best. Worst off were ‘Old’ households, where members were unable to work hard and often supported young grandchildren, while their adult children had stopped sending remittances as they had established households of their own.

**Conclusions:**

While households may adopt diverse coping mechanisms, the stage in the household life cycle when stressful events occur is important for coping outcomes. Households of the elderly and households with many young dependents are clearly vulnerable. These results demonstrate that household life cycle analysis can be useful in assessing ability to respond to stressors and shocks, including AIDS-related illness and death.

## Introduction

Rural communities in the developing world experience a spiral of stressors and shocks that cause numerous socio-economic changes during their life. These include unpredictable weather patterns that affect agricultural production and long-term political and economic instability. In many countries in Africa, the HIV/AIDS epidemic has also contributed to prime-age illness and death and labour, asset and income loss, affecting productivity and livelihoods, particularly for those in agricultural communities [1, 2]. It is estimated that it can take up to three decades before full knowledge of the effects of the epidemic on households can be obtained [3]. Over time, these different changes have compounded into ever-growing challenges to which households continuously respond in various ways, with different outcomes. Isolating the effects of a single source of stressors, such as the effect of the HIV/AIDS epidemic, can be challenging for policy-makers [4–6]. Studies that have conducted long-term monitoring still find it difficult to demonstrate the impact of HIV/AIDS and household ability to cope [4, 5, 7–10].

This study makes use of the availability of detailed longitudinal data from a study cohort in rural Uganda to analyse how the severity of the effects of adult illness and death on households can be linked to household life cycle stages. Exploring these links can give valuable insights about household vulnerability to stresses and shocks, including AIDS related challenges. The life cycle perspective recognises that as individuals in households move through their lives over time, their roles, resources, capabilities and vulnerabilities change [11]. The common family paths included in some studies are; young-singlehood, marriage, childbirth, children leaving the household, old-singlehood, death or disability of household head, and takeover by the next generation [5, 12]. In the context of the present study the life cycle concept is used to mean the stages of development that a household (as opposed to an individual) goes through over time. There can be variations and overlaps in life cycles and non-traditional household structures exist as well, such as single adults or adults who do not leave the parental home. We demarcated the stages by merging several common family cycle stages to come up with three broad stages that reflected the issues arising from the empirical material; ‘Young’, ‘Middle-aged’ and ‘Old’. The demarcations are mainly based on the presence and age of dependents, and we characterise these stages further in the analysis.

Most of the population in Uganda subsists on agricultural production through small-scale farming that is reliant on rainfall. Farms are run by households, which are defined as kinship groups that share foodstuffs and that live together or in close proximity [13]. An adult illness or a death within a household can lead to outcomes that may range from mild to severe. While some households may be able to cope well with the change and even improve their livelihoods, there are those that will struggle and experience difficulties, such as food shortages or even dissolution of the household. Household ability to cope is defined here as responding to changes resulting from illness or death so as to sustain livelihoods. In the analysis we used ‘coping well’, meaning that the household managed to meet basic needs also some time after the event, and ‘struggling’, meaning that the household either struggled to meet basic needs from the onset of the events, or that the household initially managed to cope, for example through selling assets or relying on support from outside, but in the long term was unable to meet basic needs.

Studies show that when environmental, economic, political and health-related events occur, the response strategies vary depending on the household characteristics and level of asset ownership [8, 10], factors which may also be associated with the household’s life cycle stage. There are few studies in Uganda analysing how the ‘age’ of the household influences the ability to cope and those studies that have been performed use national-level data and focus on a single age group, such as the elderly [14]. In the present study, a household life cycle perspective was used to examine differences in how individuals in households experience and respond to illness and death events. The households included were part of a study cohort in rural Uganda and hence the contextual information and established rapport and relationships necessary for this kind of analysis were already in place.

## Methodology

### Sampling

This study was nested within the Rural Livelihoods Study (RLS) [4] which was itself based within a larger General Population Cohort (GPC) study, a cohort of 5000 households from 25 villages of Kyamulibwa sub-county in Kalungu district, which has been followed for 25 years from 1989 [15]. The survey data for the GPC households were checked for death of an HIV-infected adult over the previous 20-year period and divided into two groups; those with and those without an AIDS-related death. From each of the categories, a random sample of 200 households was selected for the RLS. The first 100 households from each category were traced for interviews, while the other 100 households were used for replacing dissolved/emigrating and unwilling households. Social and economic data, such as asset ownership, cropping patterns, individual relations and income sources, were collected from households and used to examine how the epidemic had affected progress and livelihoods.

For the present study, a sub-sample of 22 households was drawn from the RLS for qualitative, in-depth analysis of household responses to the stressors and shocks caused by events of illness and death. These households were purposively selected to represent equal numbers of female- and male-headed households. In addition, the households were chosen to represent equal numbers of those affected by HIV/AIDS and non-affected households (data on HIV infection had been collected as part of the GPC). However, when in-depth data were collected, it emerged that more than half of the households selected, 16 out of 22 (72 %), had experienced long-term illness and/or death of an adult household member at some point in their recorded history. For this particular paper, we focus on the households that have experienced illness or death, which means we are analysing data from these 16 households.

### Data collection

Two local research assistants, male and female, carried out the interviews and observed changes in livelihood sources and changes in household composition and structure. This was done during monthly, two-hour visits to each household over the course of a year (2009/2010). The time was spent with the household head, spouse or any other adult household member, walking around compounds, fields and work premises, participating in activities and asking about on-going activities and recent and past events. The research assistants made notes of their visits, interviews and conversations, from which they produced detailed accounts of what was said and what they saw. The data captured events such as illness, death, droughts; sources of livelihood and livelihood activities; childcare and education; and household composition and structure. During this time, Jovita Amurwon (JA) also carried out observation visits to some households and around the study area in order to understand the current status of households and the study area. The field notes were written in English and sometimes in Luganda, which is the most common local language. All field notes were translated and transcribed by the research assistants. Additional data were collected by JA on issues that, although external to the households, nevertheless affected them, such as prices, seasonality, crop changes, drought and political events.

### Data analysis

Data analysis was performed in three phases. In the first phase, JA analysed the stories after each household visit, paying attention to emerging themes, which is a goal of induction [16–18], and identifying areas for further exploration and comparison. By the end of the 12 visits to the households, some common themes had emerged. Among these were the observation that stages in the household life cycle seemed to have a connection to the ability to cope with illness and death events, which we decided to explore further with in-depth analysis.

For this particular paper, the second stage of the analysis focused on the 16 households that had experienced extended illness or death events of adults in the past 20 years. In order to see life cycle stage-related differences between households that had experienced similar stressors, we thus decided to exclude (the few) households that had experienced death of children or stresses like drought without related extended illness/death from this stage of the analysis. All events in the households were however analysed when looking at patterns of coping in stage 3. In stage 2, JA analysed the household stories in depth to determine at what stage in the household life cycle each event had happened, and whether the household had coped well or struggled to cope with the changes brought about by the event. Three broad life cycle stages were established in order to grasp the differences that we believed to have identified in the initial analysis; ‘Young’, ‘Middle-aged’ and ‘Old’. A ‘Young’ household had acquired land through purchase or inheritance and the heads of household were usually married and had young children. In the ‘Middle-aged’ household, the children had grown up and started contributing either with labour or through sending remittances. In an ‘Old’ household, adult children had typically moved out while the head often cared for grandchildren or other younger dependents who also helped with minor household duties. A household was deemed to be ‘coping well’ if it had been able to adapt to the change and meet its basic needs over time, including e.g. food and education of children. A ‘struggling’ household was identified as one that could barely meet all its basic needs, either from the onset of the stress event or after having exhausted existing buffers.

**Fig. 1:**
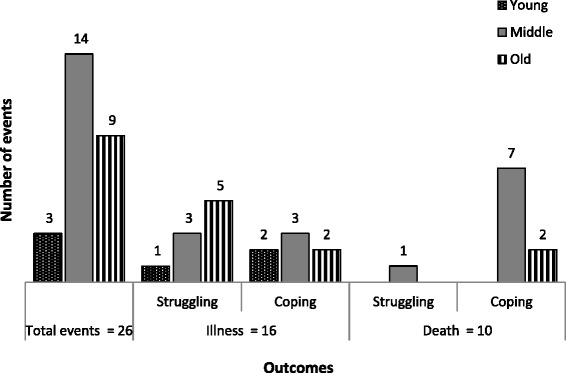
Illness and Death Events and Outcome by Life-Cycle Stage of the Household

The process of determining the stage in the household life cycle and level of coping was not always straightforward. Single denominators, such as food availability, were used to determine coping, together with a detailed qualitative analysis of household assets, responses and outcomes concerning each event. The analysis in stage 2 was focused on determining if there indeed seemed to be a correlation between household life cycle and ability to cope, and led to the plotting of these two factors against each other, in Fig. 1.

In a third analytical stage, JA looked at the rich data from the household histories of all the 16 households and identified important processes leading to coping or struggling outcomes. In this stage, all available information on these households, including death of children, weather events and many other issues were analysed. In order to illustrate the most important processes identified in this stage of the analysis, we have chosen to share three household stories that illustrate different processes in an illuminating way.

### Ethical issues

The study received ethical approval from the Science and Ethics Committee of the Uganda Virus Research Institute and the Uganda National Council for Science and Technology. Written informed consent was obtained from all participants in the study. All names used in this article are pseudonyms.

## Results

Of the 22 households interviewed, 16 households reported up to 26 separate illness (16) and death (10) events of adults, Fig. 1. Of the 16 sickness events recorded, three took place in Young households, six in Middle-aged households and seven in Old households. Of the 10 death events, eight were in Middle-aged households and two in Old households. The outcomes indicated that most households had managed to cope with illness and death, as there were 16 cases of ‘coping well’ compared with 10 of ‘struggling’. Furthermore, the analysis seemed to suggest that Middle-aged households coped more easily following a death, as seven out of eight Middle-aged households that experienced a death coped well, while five out of seven Old households struggled following an illness event. There were usually also other explanations for coping experiences, as listed in the discussion section.

### The processes underlying coping: Illustrative stories from three households

The complex processes governing coping and their relation to the household life cycle can be better understood by examining the life histories of individual households. Therefore we have selected three households that represent contrasting life cycles and coping abilities and whose stories illustrate different processes and outcomes, and present these below.

#### Young household: Mutebi’s story

Mutebi was 53 years at the time of the interviews. From the age of thirteen, he did not have access to land and instead engaged in *leja*-*leja* (meaning casual labour) on other people’s farms. When he had saved some money from this work, he started a series of income-generating activities: he sold deep-fried fish and five years later he managed to use the profits for trading in coffee and brewing alcohol from bananas. He used the income derived from this to purchase land and livestock. To construct his house, he employed builders on credit and the materials he used were borrowed from friends. He paid after he had harvested and sold coffee, alcohol and pigs. He got married in 1997 and had two children, in 2001 and 2005.

In 2000, at the age of 34, Mutebi contracted an AIDS-related illness and was bedridden for two years. He sold all his land and livestock to meet medical bills and home needs. Having lost his own fields, Mutebi utilised his father’s land for cultivation. During the same period there was a drought (from 1998), in addition to pests and diseases that caused his crops to fail. Furthermore, in 2002 Mutebi’s wife and first child became sick with an AIDS-related illness. Mutebi sold coffee to meet their medical bills but eventually illness left Mutebi too weak to ride his bicycle and he had to stop his coffee trading. During this time, he purchased bananas from the village and continued brewing alcohol for income. In 2008, Mutebi inherited land when his father died.

As Mutebi’s children were still young, he had to hire labour for agricultural work and household chores, which he paid when he harvested and sold his coffee. Following a long dry period in 2008, all the crops failed and the household experienced food shortages. Mutebi found it difficult to cope, but managed to get some food from the shop on credit and paid after harvesting coffee. From 2009, Mutebi hired out space on his compound for pig slaughtering and selling pork. He earned two kilograms of pork whenever a pig was slaughtered, which happened around twice a month. In 2009 Mutebi’s household was categorised as struggling because of the difficulties with sustaining livelihoods.

#### Middle-aged household: Ntonio’s story

Ntonio was 75 years of age when these interviews took place. He had 10 acres of land which he had acquired from his parents as a teenager during the 1950s, on which he cultivated coffee and bananas and raised livestock. He married Nanta, his second wife, in 1971, and already had four children from a first wife. Ntonio also fostered his brother and a nephew. In addition to the labour from his household, he employed three *bapakasi* (local word for ‘porters’) on the farm. By the 1980s Ntonio’s farm output was high. He sold coffee, bananas and alcohol and acquired more assets, including 3.5 acres of land and a motorcycle.

By 1990, Ntonio’s first four children had moved to the city to work. His daughter trained and found a job as a nurse. In 1993, his brother bought his own land and moved and in 1996 his nephew joined his parents in the city. While the departure of these household members resulted in loss of agricultural labour, the burden on household resources was reduced and at the same time secure livelihoods were diversified.

In 2000, Ntonio became ill and was hospitalised for three months. To cope with the costs, the household sold eight cows, the motorcycle and the stored coffee from previous seasons. During the same period, there was a long dry spell, pests and diseases that caused crop failure and death of cows. This started a vicious cycle of falling farm output, which led to less income and the inability to pay workers, which in turn led to the workers quitting. This exacerbated the falling farm output, as parts of the land had to be left fallow for two seasons. Despite this, Ntonio continued to fund his children to receive vocational training. With start-up capital from Ntonio, they started their own businesses, for example carpentry and photography in the city. As a result, resources were freed and later diverted to healthcare. This also meant that the household had some savings ahead of any other income risks or weather shocks. In 2003, Ntonio gave 3.5 acres of his land to three of his sons. One son moved to his land directly, but the others moved to the city to find work and Ntonio continued to utilise their land.

The demand for healthcare increased in 2003 because Ntonio became ill again. This coincided with a worsening of the dry spells. However, Ntonio’s household received remittances from his adult children and brothers who worked in the city, and this kept the household afloat. This income was spent on healthcare, food, educating the young children and paying for *leja*-*leja* and farm inputs such as manure and fertiliser. Pest-resistant coffee and bananas were planted, new crops like cassava were introduced and a piggery was established.

From 2006, the farm output increased: the household had plenty of food and was able to sell coffee, cassava and bananas. During the drought of 2008/2009, the household did not experience food shortages. Ntonio’s wife Nanta harvested and sold three sacks of cassava tubers each at UGX 60 000 (about 22 USD each at 2010 exchange rates), which was a significant amount of money in their village.

The household was also able to provide for more members who joined for support: three orphaned grandchildren were adopted in 2007 and 2009 and two adults, a sister of Nanta’s and a friend’s son joined the household temporarily in 2009 due to divorce and to be closer to school, respectively. In 2008, Ntonio was hospitalised again for one month and has since moved to live with his daughter (the nurse) for better care. Ntonio’s household was able to meet its basic needs and was therefore categorised as coping well in 2009.

#### Old household: Kaloli’s story

Kaloli was 82 years old at the time of the interview in 2010. In 1942, twelve-year-old Kaloli migrated to Uganda from Rwanda to work as a *mupakasi* (singular of *bapakasi*) and five years later he married. Between 1948 and 1960 he was able to manage up to seven acres of land, mostly because of the savings from his wages. In addition to subsistence crops, he also planted cash crops, including coffee, cassava, beans and groundnuts. Kaloli also fished in a nearby swamp and wove mats and fishing-baskets. By 1970, Kaloli and his wife had seven children; four boys and three girls. By 1990, the girls had married and moved away and one boy had moved to the city to find work.

Kaloli gave shares of land to two of his adult sons, which they both sold. They used the money to move to town to find work. However, they had little education and no vocational skills, so they only managed to find piece-work jobs and did not send any remittances back. Instead, Kaloli had to take care of one son when he later re-joined the household for terminal care. Kaloli’s eldest daughter divorced in 1995 and also re-joined the household with four of her children. She remarried in 1997, but left the four children with Kaloli. Two years later, she died of an AIDS-related illness. Finally, another grandson joined his household in 1996.

In that same year Kaloli’s wife fell sick and was admitted to hospital. Kaloli sold an acre of land and his stocks of coffee from previous seasons to settle the accumulated treatment costs. His wife passed away two years later, in 1998. In the following year, his son drowned in Lake Victoria. Kaloli sold another acre of land to transport the body back to the village and cover burial expenses. In 1998, after a five-year stay in Kampala, Kaloli’s second son re-joined the household in need of care. His wife had left him after he had fallen ill and he needed support, which his family provided until he died of an AIDS-related illness a few years later.

From 1998, the land that the household possessed was not enough to produce food for its ten members. Kaloli also fell sick and was too weak to hire out his labour on other people’s farms. Furthermore, during the same period there was a long dry spell and pests and diseases that caused crops to wither. The swamp also dried up and Kaloli could no longer fish. Due to lack of food, Kaloli’s four grandchildren that his late daughter had left with him moved back to their father in another village. In 2005, Kaloli’s youngest daughter re-joined the household after a divorce and brought with her four children. That daughter needed money to start a business and so the household rented out one acre of land. This money was used for training and starting a business in hair-dressing and was able to occasionally provide a 100 kg bag of maize flour.

During 2008, there was a long dry spell that caused crop failure, resulting in food shortages. Kaloli’s daughter could no longer afford to buy maize flour because the price had increased. The father of her four children gained custody because the daughter was not able to feed them. The household experienced labour shortage, not only because Kaloli was weak but also because he was unable to hire labour due to lack of money. Kaloli struggled to cultivate the land with the help of his grandson and they stopped growing labour-intensive crops such as groundnuts and millet. His grandson also carried out household chores and sometimes skipped school to do various piece-work jobs. In addition, the household had problems with infertile soils and stopped growing bananas. Because of the above listed difficulties to sustain livelihoods, Kaloli’s household was categorised as struggling in 2009.

## Discussion

In order to improve understanding of how rural-based households in a sub-Saharan African context experience the HIV/AIDS epidemic, in this analysis we investigated whether household life cycle could partly explain differing experiences of coping with the changes related to illness or death of adult household members.

The results suggest that focusing broadly on households experiencing illness and death (such as current policies targeting e.g. orphans only do) is likely to be a blunt tool for reaching the most vulnerable. The majority of the households in our sample managed to cope with illness and death events, which suggest the need for deeper understanding of the processes that enable households to cope or not. The factors that characterise households, such as life cycle stage, play an important role in determining their responses to illness and death.

Faced with effects of illness and death, household response dynamics and decision making are sometimes similar, but do not always result in the same outcomes. Some may adjust and cope well with the changes, while others may struggle to cope, either from the onset of the problems or after some time of dealing with the difficulties. The latter occurs especially when households apply practices that are detrimental to long-term livelihoods (such as selling of crucial assets) [19]. The ability of a particular household to respond to such events may be determined by several factors, including household composition, availability of labour and accumulated assets and social networks, all of which are related to what we call the ‘household life cycle’.

Here we assessed the timing of illness and death in relation to three main life cycle stages (Young, Middle-aged and Old) and found that household (in)ability to cope with change could often be better understood through this analytical approach [5, 12, 20]. Life cycle stage had implications for the nature of resources that the individuals in the household could access in order to adapt to events caused by adult illness and death.

The individual life stories pointed to links between the household life cycle and coping or struggling outcomes, but also to other important factors influencing coping. Having many adult household members meant more opportunities for diversification of livelihood activities and better chances of coping. Middle-aged households had adult children who could help with livelihood activities at home or send remittances. For example, we learned that even though Ntonio’s household was subjected to multiple stressors when he fell ill, its members managed to cope and eventually to enhance household livelihoods. This was to an extent because Ntonio had planned and invested in assets that could be sold in a crisis and in education and vocational training for his children, who were able to help the household with remittances later. However, these successful strategies were dependent on having resources available (such as land), and on having several adult children ready to start working in town when the crisis hit. Timing was crucial for this household: it had had time to prepare and build up financial and human resources, a combination which enabled it to cope with the effects of illness in an almost ideal way.

Comparison of Ntonio’s and Mutebi’s cases shows that similar experiences can produce different outcomes: both these men fell ill during a severe dry spell, when Mutebi was in his 30s and Ntonio in his 60s. While Mutebi struggled to cope with these crises, Ntonio coped well. Young households usually have many dependents that are too young to contribute significantly to livelihoods, so Mutebi’s was vulnerable in the event of illness. His household had to cope with both adults (himself and his spouse) in the family being ill at a time when the children were still too young to be of much help with livelihood activities. As a Young household, the timing of HIV infection in Mutebi’s household worked against him. In addition, unlike in Ntonio’s case, by not having inherited land and livestock, Mutebi had to devote time as a teenager to acquiring these resources. This meant that he made a late start in accumulating assets and had less of a buffer when illness hit at an early stage.

Kaloli’s household highlights two key aspects regarding coping: that owning assets and having adult children may not always result in ability to cope, and that timing of events in relation to household life cycle is certainly not the only predictor of how households will cope with crisis. We found that Kaloli’s ‘Old’ household experienced difficulties with hard labour and supported young grandchildren, just as Baylies [21] has also noted in cases of older households. By this stage of the household life cycle, the adult children of the Old households in our sample had often stopped sending remittances after establishing households of their own. In some cases many of the Old households had already passed on land and other assets to their children. Therefore, decisions regarding assets, relationships with adult children and the strength of a household’s social network were all crucial to coping. Kaloli’s adult children did not send remittances to help the household when the crisis hit. Instead, they drained resources from the household when they sold land, sent children needing care to the household or re-joined the household when needing care upon ill-health. The concept of ‘coping strategies’ may be misleading, as responses by individuals to changes brought about by adult illness and death sometimes result in exacerbating the situation [19]. In some circumstances, households and individuals may successfully apply conscious and strategic planning and adaptation. In other instances, however, they are unable to plan, react sub-optimally or are in various ways constrained from applying good plans in practice.

Thus studying the household life cycle can be a useful analytical approach in assessing the ability of households to respond to stresses and shocks, including AIDS-related illness and death, in rural sub-Saharan Africa but also elsewhere. Other aspects of vulnerability, such as being AIDS-affected or orphaned, disabled, widowed or divorced, male or female, and different levels of wealth and power, however also have profound effects on coping, as do of course various policies targeted at households, such as universal access to free ART [22]. The household life cycle should thus be treated as an additional analytical tool and not as a full explanatory framework. However, we believe that analysis of life cycle stage is a useful addition to the various perspectives attempting to analyse and explain why some households cope well while others struggle when affected by prolonged illness or death.

Future studies could further investigate the link between household life cycle and coping through including a larger sample of cases and looking at potential impacts of policies targeted at households with young children and elderly people. Policy-related conclusions should however be drawn with caution, as household experiences of HIV/AIDS are not homogeneous and there is no direct connection between AIDS-related events and various changes observed in households [4, 5, 7, 23].

## Conclusions

The timing of illness and death events in relation to a household’s life cycle is a relevant factor among others in determining the household’s capacity to cope with these changes. At different stages in the life cycle, the experience of and response to illness and death of adults vary and may result in different socio-economic outcomes for the household. This is due to variation in the characteristics of the household and the nature of resources to which individuals have access over time. A household in the Middle-aged stage often has a better capacity to cope due to availability prime-age labour and accumulated social and physical assets such as social networks, land and business investments. The vulnerability of the elderly is mostly due to loss of assets and ability for labour, while young households usually have young dependents and have not yet accumulated assets.

## Abbreviations

MRC: Medical Research Council
UVRI: Uganda Virus Research Institute
GPC: General Population Cohort
RLS: Rural Livelihoods Study
